# ‘*Candidatus* Liberibacter Solanacearum’ Is Unlikely to Be Transmitted Spontaneously from Infected Carrot Plants to Citrus Plants by *Trioza Erytreae*

**DOI:** 10.3390/insects11080514

**Published:** 2020-08-08

**Authors:** María Quintana-González de Chaves, Gabriela R. Teresani, Estrella Hernández-Suárez, Edson Bertolini, Aránzazu Moreno, Alberto Fereres, Mariano Cambra, Felipe Siverio

**Affiliations:** 1 Departamento de Protección Vegetal, Instituto Canario de Investigaciones Agrarias (ICIA), Crta. El Boquerón s/n, 38270 La Laguna, Spain; ehernand@icia.es (E.H.-S.); fsiverio@icia.es (F.S.); 2 APTA-Instituto Agronômico (IAC)-Centro de Pesquisa e Desenvolvimento de Fitossanidade, Campinas 13020-902, Brazil; gabiteresani@gmail.com; 3 Faculdade de Agronomia, Departamento de Fitosanidade, Universidade Federal do Rio Grande do Sul (UFRGS), Avenida Bento Gonçalves 7712, Porto Alegre 91540-000, Brazil; edson.bertolini@ufrgs.br; 4 Instituto de Ciencias Agrarias, Consejo Superior de Investigaciones Científicas (CSIC), Calle Serrano, 115, 28006 Madrid, Spain; amoreno@ica.csic.es (A.M.); afereres@ica.csic.es (A.F.); 5 Instituto Valenciano de Investigaciones Agrarias (IVIA), Centro de Protección Vegetal y Biotecnología, Carretera CV-315, Km 10.7, 46113 Moncada, Spain; mcambra@mcambra.es; 6 Sección de Laboratorio de Sanidad Vegetal, Consejería de Agricultura, Ganadería y Pesca, Gobierno de Canarias, Ctra. El Boquerón s/n, 28270 La Laguna, Spain

**Keywords:** vector behavior, psyllids, transmission vector-plant-pathogen interactions, EPG, oviposition, dodder, budding, feeding

## Abstract

**Simple Summary:**

The potential transmission of the bacterium ‘*Candidatus* Liberibacter solanacearum’ from infected carrot plants to citrus plants by the African citrus psyllid (*Trioza erytreae*) should be considered and therefore studied, because this psyllid is an efficient vector of citrus huanglongbing disease (associated to bacteria from the same genus). The aim of this study was to assess the bacterium transmission by three different ways: dodder, grafting and the African citrus psyllid. Additionally, the feeding behavior and oviposition of this psyllid were also evaluated. The bacterium was only transmitted from carrot plants to citrus plants through dodder, although the infection was not established. The African psyllid could settle and oviposit in carrot plants, but it was not able to complete its life cycle on them. This psyllid acquired and transmitted the bacterium from carrots to carrots but was not able to transmit it to citrus plants. In conclusion, after having assessed all relevant possibilities by experimental transmissions from infected carrot plants to citrus plants, the bacterium was transmitted but not established. Our data suggest that the bacterium transmission to citrus plants by the African citrus psyllid is unlikely.

**Abstract:**

Bacteria belonging to ‘*Candidatus* Liberibacter spp.’ are associated with various severe diseases in the five continents. The African citrus psyllid *Trioza erytreae* (Hemiptera: Triozidae) is an efficient vector of citrus huanglongbing-HLB disease, absent in the Mediterranean basin. This psyllid is currently present in the islands and mainland Portugal and Spain, where the prevalence of ‘*Ca.* Liberibacter solanacearum’ (CaLsol) associated to a carrot disease is high. *Trioza erytreae* normally feeds on citrus plants but has also been observed on other crops. It would be a great concern to the Mediterranean citrus industry if *T. erytreae* could transmit this bacterium from carrots to citrus and cause disease; therefore, the transmission of CaLsol from carrot plants to citrus plants was experimentally assessed. Although CaLsol was initially detected on receptor citrus plants in transmission assays by dodder and budding, the infection was not established. The feeding behavior by electrical penetration graphs and oviposition of *T. erytreae* on carrot plants versus citrus plants was evaluated. *Trioza erytreae* only reached the phloem in citrus plants. However, it was able to acquire CaLsol from infected carrots but unable to transmit it to citrus plants. CaLsol was detected in some carrot plants immediately after 7 and 14 days (inoculation access period), but it was not detected after one month. *Trioza erytreae* was unable to complete its life cycle on carrot plants. In conclusion, the efficient vector of bacteria associated to huanglongbing was unable to transmit CaLsol from carrot to citrus plants, but it acquired and transmitted the bacterium from carrot to carrot plants with low efficiency.

## 1. Introduction

‘*Candidatus* Liberibacter solanacearum’ (CaLsol) is a phytopathogenic bacterium associated with aggressive diseases in Solanaceae and Apiaceae crop species. This bacterium was reported for the first time in potato (*Solanum tuberosum* L.) associated to zebra chip disease in the USA as ‘*Candidatus* Liberibacter psyllaurus’ [1] and then in New Zealand as CaLsol [2,3]. This bacterium also affects tomato (*Solanum lycopersicum* L.) [1], pepper (*Capsicum annuum* L.), Berlandier’s wolfberry (*Lycium berlandieri* Dunal), tamarillo (*Solanum betaceum* Cav.), eggplant (*Solanum melongena* L.) [4], tobacco (*Nicotiana tabacum* L.) [5], dulcamara (*Solanum dulcamara* L.), *Solanum elaeagnifolium* Cav., golden berry (*Physalis peruviana* L.), *Solanum americanum* Mill. and goji berry (*Lycium barbarum* L., *L. chinense* Mill.) [6]. In Europe, this bacterium was associated for the first time with carrot (*Daucus carota* L.) disease in Finland [7] and then in Spain [8,9]. It has since been found in celery (*Apium graveolens* L.) [10], parsnip (*Pastinaca sativa* L.), parsley (*Petroselinum crispum* Mill.) [11], chervil (*Anthriscus cerefolium* Hoffm.) and fennel (*Foeniculum vulgare* Mill.) [12].

The bacterium is naturally transmitted by psyllid (Hemiptera: Triozidae) vector species. Eight different haplotypes of CaLsol have been found in different areas. Haplotype A and B is transmitted by the psyllid *Bactericera cockerelli* (Šulc, 1909) and affects Solanaceae in North and Central America and New Zealand (only haplotype A) [4,5,6,13]. Haplotype C is transmitted by *Trioza apicalis* Foerster, 1848, generally affecting carrot crops in North Europe [5,7,14,15], but it has been found in symptomless field-grown potato in Finland [16] and in *Trioza anthrisci* Burckhardt 1986 [17]. Haplotype D and E is transmitted by *Bactericera trigonica* Hodkinson and affects Apiaceae in South Europe and North Africa [11,12,15,18,19,20,21,22]. Haplotype U was detected in *Trioza urticae* (Linné, 1758) in Finland [23], haplotype F was found in one potato tuber in the USA [24], and haplotype G and haplotype H are the most recently discovered in USA and Finland, respectively [25,26].

To date, *B. cockerelli*, *T. apicalis* and *B. trigonica* are the main insect host vector species of CaLsol associated to several diseases [1,3,7,9]. However, other psyllid species, such as *Bactericera nigricornis* (Foerster, 1848) could be potential vectors of CaLsol to new host [27,28,29]. *Trioza erytreae* (Del Guercio, 1818) has been found on other plant species, such as overwintering plants, food plants or casual plants, on which it can survive, feed or land [30]. In fact, although Rutaceae species (mainly *Citrus* spp.) are the main hosts of *T. erytreae*, it has been frequently seen visiting and landing in carrot crops and vineyards in the Canary Islands (Supplementary Material, Figure S1 and Video S1), among other crops and weeds.

Moreover, the most destructive citrus disease, Huanglongbing (HLB), is associated to the genus ‘*Candidatus* Liberibacter spp.’ specifically the species ‘*Ca*. L. africanus’ (CaLaf), ‘*Ca*. L. americanus’ (CaLam) and ‘*Ca*. L. asiaticus’ (CaLas). The African citrus psyllid (*T. erytreae*) is one of the main vectors of HLB disease. Although this psyllid species has been present in Madeira [31] and the Canary Islands since 2002 [32] and along the North-western and central Atlantic coast of Portugal and in North-western Spain, in the Iberian Peninsula, since 2014 [33], HLB has never been detected in Europe [34]. The spread of this psyllid species to the main citrus-producing areas is one of the greatest concerns of the economically and socially important Spanish and Mediterranean citrus industry. In fact, in Spain, three national contingency plans were published to avoid the entry and spread of the main psyllid vector species *Diaphorina citri* Kuwayama, 1808 (Hemiptera: Liviidae) and *T. erytreae*, as well as the bacteria associated with HLB (mentioned above) [35,36,37]. In addition, the Mediterranean region is the largest citrus-producing area in Spain, where this crop coexists with other vegetable crops such as carrots, frequently with a high prevalence of CaLsol [8,9,15,28,29,34,36,37].

The study of potential vectors of CaLsol and its transmission to new hosts is essential in order to answer key questions related to epidemiology. Such as: Could CaLsol be spontaneously transmitted to citrus plants? Are there any potential psyllid vector species that can transmit the bacterium from frequently infected carrot to citrus plants? The consequences of the hypothetical transmission of CaLsol to citrus are unknown, but the economic losses caused by ‘*Candidatus* Liberibacter spp.’ associated with HLB, which is well established in affected countries, must be taken into account since the rapid and aggressive spread of HLB has disrupted the citrus industry in recent decades [38,39]. This possibility could put the main citrus-producing areas of Europe and Mediterranean basin at risk. It is uncertain whether *T. erytreae* can occasionally feed on CaLsol-infected carrot plants, acquire the bacterium and transmit it to citrus plants or even if CaLsol is able to thrive on rutaceous plants.

In this work, the transmission of CaLsol to citrus plants was studied through three experimental and natural ways of transmission: dodder, grafting by budding and by the psyllid *T. erytreae*. In addition, the oviposition and feeding behavior of *T. erytreae* was evaluated in order to assess the probability of transmission of CaLsol from infected carrot plants to citrus plants.

## 2. Materials and Methods

### 2.1. Source of Insects

All the individuals used in the different studies belong to the ad hoc colony established in the insect-proof facilities of the Instituto Canario de Investigaciones Agrarias (ICIA). Individuals of *Trioza erytreae* were originally collected from sweet orange trees in the municipality of Tegueste (Tenerife, Canary Islands). This colony was maintained on two-year-old pesticide-free citrus plants: lemon (cv. Eureka grafted on *Citrus macrophylla*) and sweet orange (cv. Lane Late IVIA 188 on Citrange Carrizo). Plants growing in pots (Ø = 20 cm) and grown under controlled conditions (20 ± 5 °C, RH > 70%, 16:8 h (L:D) photoperiod). These citrus plants were regularly pruned to stimulate the emergence of new shoots. Adults from this colony were often tested by real-time PCR [10] to confirm the healthy status in relation to CaLsol.

### 2.2. Plant Material

Plant material of each assay was certified pesticide-free and was kept in pots under controlled conditions (20 ± 5 °C, RH > 70%, 16:8 h (L:D)) inside a mesh cage on insect-proof facilities. Plants were analyzed by real-time PCR according to the protocol designed by Teresani et al. [10].

Carrot Bangor F1 was the cultivar used in all assays and plants were obtained from seeds (Bejo Iberica S.L.U. LOT 1098592).

#### 2.2.1. Donor Plants

CaLsol inoculum (donor plants) were symptomatic infected carrots (CaLsol haplotype E) collected from the field (same locality as above) (except budding transmission). CaLsol positive citrus plants from dodder transmission assay (see below Section 2.4) were used in budding transmission studies (see below Section 2.5).

#### 2.2.2. Receptor Plants

In the experimental transmission studies through dodder (see below Section 2.4), the receptor plants were carrots (10–15 leaf stage) and two-year-old sweet orange (*Citrus sinensis*), Mexican lime (*C. aurantifolia*) and rose periwinkle (*Catharanthus roseus*).

In the transmission studies by budding (grafting) (see below Section 2.5), the receptor plants were two-year-old sweet orange (cv. Pineapple) and Mexican lime.

Young sour orange seedlings (*Citrus x aurantium*) (7–10 leaf stage) were used in feeding behavior assay (see below Section 2.6).

Carrots (5–10 leaf stage) and two-year-old sour orange plants were used in CaLsol transmission studies by *T. erytreae* (see below Section 2.7).

#### 2.2.3. Plant Material in Setting and Oviposition Studies

Sour orange plants from seeds (eleven-week-old) and carrots (six-week-old) in pots (Ø 5 cm) were used in setting and oviposition assays.

### 2.3. DNA Extraction, Detection and Haplotyping of CaLsol

Samples of leaves or plant shoots were collected and stored at 4 °C and/or −20 °C until analysis. The plant samples were homogenized using a Homex 6 homogenizer (Bioreba, Reinach, Switzerland, CH) in PBS extraction buffer at 1:10 (*w/v*) (pH 7.4). Total DNA was obtained from 200 µL of crude plant extracts using a modified cetyl-trimethylammonium bromide (CTAB) protocol without β-mercaptoethanol [40]. The extracted DNA was preserved until use at −20 °C.

Recently captured insect specimens or those previously preserved in 70% ethanol, were individually squashed on membranes and subsequently dried [10]. The membranes harbouring squashed psyllids were carefully cut out around the area where the psyllid was squashed (immobilized on the membrane) and placed into a 1.5 mL microtube with 100 µL of distilled water, vortexed and centrifuged [10]. Three microlitres of this extract were used directly for real-time PCR analysis.

A real-time PCR complet kit, CaLsol/100 (Plant Print Diagnòstics S.L. Valencia, Spain), was used for the detection of CaLsol according to the manufacturer’s instructions in conjunction with recommended standards by EPPO-PM 7/143 [40]. Nine µL of the prepared master mix provided by the kit were transferred to each PCR tube or microplate well, and 3 µL of the DNA extracts sample from plants or insects, obtained as indicated above, were added for the amplification. A StepOne Plus Real-time PCR thermocycler (Applied Biosystems) was used. Healthy plants, CaLsol noncontaminated psyllids, PCR master mix and virgin pieces of the membrane were simultaneously processed and analyzed as negative controls.

The CaLsol haplotype was determined according to Nelson et al. [5] and Bertolini et al. [41].

### 2.4. CaLsol Transmission Studies by Dodder

Vascular connections using dodder (*Cuscuta campestris*) were kept for 60 days between donor and receptor plants, according to Bertolini et al. [41], in a P2 containment level greenhouse. Five receptor plants from each species were used. The dodder was also grown on the same receptor plant species as the healthy controls. Visual inspection of symptoms was performed on a monthly basis. Samples of leaves from shoots that were not connected to dodder were collected every 30 days until six months after connection and subsequently tested for CaLsol as described previously (Supplementary Material, Figure S2).

### 2.5. CaLsol Transmission Studies by Budding Grafting

Budding assays were made according to standard procedures [42,43]. Two buds per plant in five replicates of each species were used (total 10 replicates). Buds were collected in receptor plants from transmission assays by dodder, in the immediate proximity of the leave that tested positive sixteen hours after the CaLsol detection (Supplementary Material, Figure S3).

The grafted plants were grown in a P2 greenhouse during 12 months postbudding inoculation, in order to monitor potential symptoms (fortnightly) and analyze the detection of CaLsol by real-time PCR. Several samples per plant was collected: bark material close to the inoculation site (graft) and midribs of leaves from all the shoots of the inoculated plant.

### 2.6. Feeding Behavior of T. erytreae

The probing and feeding behavior of *T. erytreae* on sour orange and carrot plants were monitored using the electrical penetration graph (EPG) technique [44] according to Antolínez et al. [45,46]. EPG recordings were obtained with a DC-EPG device (Giga-4; EPG Systems, Wageningen, The Netherlands), adjusted to a 100× gain. The monitoring system was assembled inside a Faraday cage (100 × 100 × 70 cm) to prevent electrical noise. EPG data acquisition was recorded using Stylet+ software for Windows (EPG Systems, Wageningen, the Netherlands). A total of 68 EPG recordings in citrus and 64 in carrots were made. Only those EPG recordings with an optimal signal quality were considered for the analysis: 14 and 11 EPG recordings from citrus and carrots, respectively.

Insect probing and feeding behavior were monitored for 8 h in the laboratory starting immediately after the insects were placed on the leaf. The EPG waveforms previously described for psyllid species [46,47,48] were identified as follows: nonprobing (np), intercellular apoplastic stylet pathway (C waveform), initial contact with phloem tissue (D waveform), salivation into phloem sieve elements (E1 waveform), passive phloem sap uptake (E2 waveform) and active intake of xylem sap (G waveform). Fourteen replicates were recorded for sour orange plants and eleven for carrot plants. Each replicate (individual psyllids and plants) was tested using a different plant and psyllid for each EPG recording. All behavioral variables were processed using an MS Excel workbook for automatic EPG data calculations according to the methods of Sarriá et al. [49].

The number of events and total duration of events per insect (means ± SEs) of selected EPG variables were calculated using the SPSS statistical software package according to Backus et al. [50]. The following parameters were used: the number of waveform events per insect (NWEI), which is the total number of events of a particular waveform divided by the total number of insects under each treatment; the waveform duration (s) per insect (WDI), which is the total duration of each event of a particular waveform made by each individual insect that produced that waveform divided by the total number of insects under each treatment; and the waveform duration (s) per event and per insect (WDEI), which is the sum of the mean duration of the events of a particular waveform made by each individual insect divided by the total number of insects.

### 2.7. CaLsol Transmission Studies by T. erytreae

#### 2.7.1. CaLsol Acquisition Studies by *T. erytreae*

Three trials of CaLsol acquisition by *T. erytreae* from infected carrot plants were carried out. The data are summarized in Table 1, showing the number of psyllids used per test, number of repetitions and times of acquisition in carrot plants infected with CaLsol. Psyllids which were not exposed to infected carrot plants were used as a control (10 psyllids per repetition).

#### 2.7.2. CaLsol Inoculation Studies Using *T. erytreae* as a Vector

Adult psyllid individuals were allowed to feed on CaLsol-infected carrot donor plants for an acquisition access period (AAP) of 72 h [45]. It was found that it was enough time to infect 46% of psyllids exposed to infected carrot plants (see Section 3.4.1. *Trioza erytreae* acquisition assays). All experiments were performed in a chamber under controlled conditions (20 ± 5 °C, *RH* > 70%*,* 16:8 h (L:D)). Calsol-free carrot plants without psyllid exposure were used in all the experiments as controls.

After the AAP, *T. erytreae* individuals were placed into a plastic clip cage (Ø = 3.5 cm × 4 cm high) clamped onto a leaf, where they stayed for different inoculation access periods (IAP) of 24 h, 3 days, 7 days and 14 days or until insect death (five psyllid adults per clip cage/leaf and four clip cages per plant, for a total of 20 psyllid species per plant). The assays were carried out for ten replicates per plant species (using carrot and sour orange plants as receptor plants) (Supplementary Material, Figure S4). After each IAP, the insects were collected, and the leaf was removed and cleaned with sterile distilled water. The psyllid species and leaves were subsequently tested for CaLsol by real-time PCR. Afterwards, the plants were sprayed outside the chamber with a systemic insecticide (1 g/L Confidor^®^, Bayer CropScience) and then moved to an insect-proof greenhouse, where they grew for one month. The plants were then monitored for disease symptoms, and newly developed leaves from each plant were tested by real-time PCR to assess CaLsol infection.

### 2.8. Settling and Oviposition of T. erytreae

Settling and oviposition of *T. erytreae* was monitored in twelve carrots and twelve citrus plants. A group of homogeneous adults of *T. erytreae* at fertile stage was chosen from the colony (described in 2.1 Source of insects), and each female was found to be capable to laying eggs. A pair (1♂ + 1♀) were randomly placed in each plant inside a plastic cage (Ø = 5.5 cm × 15 cm high), with a ventilation hole covered with a mesh, to prevent the escape of the psyllids and to favor the air flow. They were confined for 3 days, after this period, the pair was removed, and the number of eggs and their evolution were registered for 28 d.

### 2.9. Statistical Analysis

Statistical analyses were performed regarding feeding behavior and oviposition assays. The analysis of the data was conducted in both cases using the SPSS Statistics version 22 software (IBM).

All behavioral variables obtained by the EPG recordings were transformed prior to analysis by either the sqrt (x + 1) or ln (x + 1) and checked for normality using the Shapiro–Wilk W test. Comparison between treatments were made by Student’s t-test (Gaussian variables) or by the Mann–Whitney U test (for non-Gaussian variables). P-values below 0.05 were statistically significant.

The data obtained from the oviposition assay were checked for normality with Shapiro–Wilk W test, obtaining non-Gaussian variables. Thus, comparisons between treatments were made by Mann–Whitney U test.

## 3. Results

### 3.1. Transmission Studies by Dodder

After 3 months of phloem connection by dodder, CaLsol presence was detected in each receptor species tested. Two species of receptor plants, carrot and rose periwinkle, showed characteristic CaLsol symptoms. In carrot, red and yellow leaves discoloration and stems with multiple sprouts were observed; in rose periwinkle plants, yellow discoloration of the leaves and a decreased development/dwarfing were noticed. The bacterium was detected in both carrot and rose periwinkle plants at 6 months after dodder removal. During 3 months after dodder removal, citrus plants did not show symptoms of diseases and showed healthy growth. New citrus sprouts analyzed after 4, 5 and 6 months gave negative results for CaLsol. The haplotype of the experimentally infected receptor plants was the same as the original one in the donor plants.

### 3.2. Transmission Studies by Budding Grafting

CaLsol was never detected in new sprouts of citrus plants (both assayed botanical species) at the end of the experimental period of 8 months after challenging by budding. No symptoms of diseases were observed neither during the assay nor at 12 months’ postassay.

### 3.3. Feeding Behavior of T. erytreae

The results of the feeding behavior study for 8 h are summarized in Table 2. There were significant differences in the time to first probe from the start of the EPG duration, which was higher in the carrot plants (9804.8 ± 3786.7 s) than in the citrus plants (1649.7 ± 938.9 s). Although the number of nonprobe waveforms was significantly higher in citrus plants (25.9 ± 4.7 s) than in carrot plants (6.8 ± 1.9 s), the duration of nonprobing events was higher in the carrot plants (WDI: 23,769.7 ± 2051.2 s; WDEI: 10,748.4 ± 3608.2 s) than in the citrus plants (WDI: 14,342.0 ± 1655.9 s; WDEI: 1056.2 ± 362.5 s). The number of probes by *T. erytreae* was significantly higher for the citrus plants (25.3 ± 4.7) than for the carrot plants (5.9 ± 1.9), similarly to what happened for the duration of probes per insect (WDI: 14,431.6 ± 1655.1 s in citrus vs. 5030.3 ± 2051.2 s in carrots). Only 6 psyllids feeding on citrus plants were able to contact the phloem tissues (PPW D: 6/14), while no phloem activities were observed for psyllid species feeding on carrot plants. In citrus, the proportion of psyllid individuals salivating into the phloem cells (E1) was 28.6% (4/14), and three of fourteen (21.42%) individuals analyzed reached the continuous phloem ingestion phase (E2) during the recording period. Three of these 4 individuals reached phloem sieve elements in citrus plants, reaching phloem ingestion phases six times, and five of these six E2 phases were longer than 10 min. Therefore, 83.3% of all phloem ingestion contacts were successful.

### 3.4. CaLsol Transmission Studies by T. erytreae

#### 3.4.1. *Trioza erytreae* Acquisition Assays

The results of the acquisition assay are shown in Table 3. CaLsol was detected in 6.7%, 14.4%, 20.0%, 46.0% and 33.3% of the *T. erytreae* adults after 12 h, 1 day, 2 days, 3 days and 5 days of exposure to infected carrot plants, respectively.

#### 3.4.2. CaLsol Inoculation Studies Using *T. erytreae* Species as a Vector

The results of CaLsol detection on *T. erytreae* for the clip cage plant assay are shown in Table 4. The bacterium was never detected in either psyllid or plant controls. Psyllid species found on plants after each IAP were analyzed, revealing 14.3 to 33.3% of CaLsol-positive individuals.

CaLsol was detected in one out of the six tested carrot plants exposed to *T. erytreae* after 7 days and 14 days of IAP (inoculation access period). Nevertheless, the bacterium was never detected in citrus plants after the four assessed IAPs.

### 3.5. Setting and Oviposition Studies of T. erytreae

The *T. erytreae* survival percentages on carrot and citrus plants at 72 h after confining were 91.7% and 100%, respectively. The number of eggs laid per *T. erytreae* female per plant (mean ± SE) at 72 h was 145.75 ± 30.52 in citrus and 6.92 ± 3.49 in carrots. The accumulative number of eggs laid by *T. erytreae* was significantly higher on sweet orange plants (1470 eggs) than on carrot plants (77 eggs). On the citrus plants, 11.9% of the *T. erytreae* eggs were able to complete their life cycle until the adult stage, but none were able to complete their life cycle on the carrot plants. In total, 42.8 % of *T. erytreae* eggs survived until the N1–N3 stage on carrot plants, and the highest mortality (100%) occurred from N1–N3 nymphs to N4–N5 nymphs.

## 4. Discussion

Three different ways of transmission of ‘*Candidatus* Liberibacter spp.’: dodder, grafting and psyllids, were evaluated. However, the main way to transmit members of this genus of bacteria under natural field conditions is through psyllid vector species after introduction of infected plants. The behavior of *T. erytreae* was monitored in citrus and carrot plants, consequently the oviposition and feeding behavior was evaluated, as well as the acquisition and inoculation of the bacterium in both plant species, which has not been reported before.

The use of dodder and budding, or other grafting procedures, as transmission ways is common [16,41,51,52,53,54]. The aim of our assays was to qualitatively evaluate the transmission, following international standard procedures to design the experiments [42,43], where the use of less than ten replicates is widely accepted. In our experiment with dodder, CaLsol was transmitted from infected carrots to citrus, rose periwinkle and carrots. To our knowledge, this is the first time that the bacterium was detected in citrus plants after experimental transmission. Carrot and rose periwinkle showed characteristic CaLsol symptoms; however, positive citrus were always asymptomatic, and the bacterium was not detected several months after disconnection from the dodder. This fact may be explained because CaLsol is being transmitted to citrus plants while the dodder was establishing vascular connections with infected carrot but apparently is unable to multiply itself in citrus after dodder disconnection, at least under the conditions of our experiment. In the transmission studies by budding, CaLsol was not detected in the receptor citrus species at 8 months postinoculation by grafting. As far as we know, citrus plants were positive while they were connected through dodder to infected carrots. Once the dodder connection was removed, they were still positive before budding performance. However, CaLsol were not detected after three months of dodder removal. That is why, as authors, we believe that the CaLsol infection in citrus plants was not systemic. In a previous paper by Haapalainen et al. [16], the transmission of CaLsol haplotype C through dodder and grafting onto potato was performed, but the transmission rate was very low; the bacteria did not colonize neither the root tissues nor the tubers. The reasons remain unknown, but they could be related to the specificity of the CaLsol haplotype, which could have different interactions with alternative hosts but also the cultivar assayed could be more resistant than others used in America [16].

This study evaluates, for the first time, the feeding behavior of *T. erytreae* by EPG in citrus and carrot plants, although this characteristic has been studied in other nearby species, such as *T. apicalis*, *B. cockerelli*, *B. trigonica* and *B. tremblayi* and for other vectors of HLB, such as *Diaphorina citri* [45,55,56,57,58,59]. It is worth emphasizing that *T. erytreae* feed readily on citrus plants because four individuals reached the phloem in citrus, and for five times, the duration of each event was longer than 10 min; however, in our EPG assay there were no significant differences in the number or duration of E1 and E2 in citrus and carrot plants. The little difference between these EPG event durations may be influenced by citrus species, leaf age and EPG recording time used. A fully young expanded leaf of sour orange seedlings (which facilitates EPGs) was used, but it has been shown in previous studies that the leaf stage of citrus plants plays an important role in feeding behavior and overall in the case of *T. erytreae*, which has a strong preference to feed on young sprouts [60]. Studies carried out with *D. citri* showed that it prefers to ingest from the phloem of immature leaves but from the xylem of mature leaves [59]. The citrus species used in our assay was sour orange, a frequent host of *T. erytreae* in the North of the Iberian Peninsula (Portugal and Spain) as ornamental tree in private and public gardens, where damages and frequency of infestation by this pest have been previously reported [34]. Additionally, this species is commonly used as a rootstock for lemon and as a crop for the jam industry. All in all, it would be interesting to perform new feeding behavior assays with other citrus species, such as lemon, sweet orange or mandarin trees, since certain preference of *T. erytreae* have been seen in the fields where the most frequently grown citrus species are: lime, lemon, mandarins, sweet orange and grapefruit trees [60,61,62]. For our study, eight hours of EPG recording time was used as this is the standard time used in many previous similar EPG studies, e.g., in those involving *B. trigonica*, *B. tremblayi* and *D. citri* [45,54,63]. However, EPG recordings of *B. cockerelli* were documented for 24 h [55,64] and in *D. citri* for 42 h [58]; if shorter EPG recording times are used, it could be problematic to quantify the first phloem ingestion phase by some psyllids [59]. These factors could explain the low activity of *T. erytreae* reported in our assays on citrus and carrot plants. EPG is a useful technique to determine whether this psyllid species is able to reach the phloem sieves in carrot plants because this fact has great importance in the acquisition and inoculation of CaLsol. Nevertheless, this transmission has never been recorded in the Canary Islands field conditions. In routine analyses performed on a great number of citrus plants and *T. erytreae* individuals, using the universal protocol to detect ‘*Ca*. Liberibacter spp.’ by real-time PCR (which also detect CaLsol and associated bacterial species to HLB), no case has ever been found [34].

The results of our CaLsol transmission studies by *T. erytreae* showed that this psyllid was able to acquire the bacterium from symptomatic carrot plants under no-choice conditions in an AAP of 72 h. We found that it was enough time to infect 46% of psyllids exposed to infected carrot plants and this result agrees with Antolinez et al. [45] who also used 72 h as AAP. CaLsol was detected in one carrot plant after 7 days and in another one after 14 days of IAP. Consequently *T. erytreae* was able to transmit CaLsol from carrot to carrot plants when it was forced to feed off of them. Nevertheless, under multiple choice conditions, which are actually like more real situations in the field, *T. erytreae* is unlikely to transmit the bacterium from one carrot plant to another because this species is not a host (*T. erytreae* is not able to complete its life cycle on this species, although it can land or feed off of carrot plants). This fact could only occur sporadically; several reports have described rutaceous plants as the only hosts of *T. erytreae* [34,60,61,62,65,66,67]. In addition, previous studies using sampling methods at the level of the crop canopy have shown that the main psyllid species present in carrot fields is *B. trigonica*, followed by *B. nigricornis* in low proportions [27,29]. Several previous studies on bacterial transmission by psyllid species in nonhost plant species found that: *T. apicalis* (a vector of CaLsol in Apiaceae species in northern Europe) does not transmit the bacterium from carrot to potato [16], *B. trigonica* (a vector of CaLsol in Apiaceae species in southern Europe and North Africa) was able to transmit CaLsol at a low rate from carrot to potato or tomato [45,54], and the transmission of CaLsol to carrots by *B. cockerelli* (vector of CaLsol in Solanaceae species) was possible at a low rate and induced disease symptoms [62]. On the one hand, *B. tremblayi* could acquire CaLsol from infected carrot plants, but it could not transmit the bacterium to carrot plants, which might be because the bacterium was not able to complete circulation in the body of the psyllid to be ultimately inoculated from the salivary glands [45]. On the other hand, Teresani et al. [54], who investigated CaLsol transmission by *B. trigonica* from carrot to potato and tomato, reported a high detection of CaLsol in the leaves immediately after the insects were removed, which may be due to inoculation in phloem sieves, contamination of the tissue by psyllid activities or detection of nonviable bacteria. A similar situation could have happened with the detection of CaLsol in our assay.

Another part of the *T. erytreae* behavior study is the oviposition on carrot and citrus plants. If *T. erytreae* is able to settle and oviposit on carrots plants, the risk of transmission would not be negligible. *T. erytreae* showed much higher oviposition on citrus plants than on carrot plants. The insects were able to survive (91.7% of adults) and oviposit for 72 h on carrot plants and 42.8% of 77 laid eggs developed until the nymph N1–N3 stage. In citrus, 11.9% of the laid eggs completed their life cycle and reached the adult stage. According to Catling [65], a high mortality of different juvenile stages in host plants was previously reported. Taken together, these results indicate a high survival of *T. erytreae* on a nonhost plant species, which contrasts with the results of previous studies conducted with *B. trigonica*, which was unable to lay eggs on tomato plants, and only five eggs were laid by a total of three pairs in potato plants, where the few hatched nymphs died during the first instar [54]. In the case of *B. tremblayi*, although it could settle and oviposit on carrot plants, it clearly preferred to oviposit in leek (its main host) [45]. A previous study of *T. erytreae* feeding preference in Kenya showed that many other rutaceous plants are alternative hosts but feeding on them could affect the morphometry of psyllids [67]. Although no studies have yet been carried out on psyllid species to establish the relation between oviposition and long-term survival on alternative hosts, our results again might confirm that psyllid species can sporadically settle in different plant species, which may help to sustain survival when the main hosts are rare or not present [30,45].

## 5. Conclusions

*T. erytreae* was assessed as a sporadic visitor of carrot plants where it is able to feed (in forced conditions of nonchoice), as shown in our experiments. Furthermore, there is no evidence and nor is it feasible that dodder would be able to colonize carrot plants and citrus plants at the same time, and it is not feasible that budding or any grafting procedure could be performed between CaLsol infected carrot plants and citrus plants. In conclusion, after having assessed all relevant possibilities by experimental transmissions of CaLso from infected carrot plants to citrus plant, the bacterium was transmitted but not established. Therefore, our data suggest that CaLsol is unlikely to be transmitted under natural field conditions from carrot plants to citrus plants.

## Figures and Tables

**Table 1:** CaLsol acquisition access period (AAP) by *T. erytreae* in infected carrot plants.

Assay No.	Psyllid No.	Repetition No.	Acquisition Period Evaluated
1	10	4	1 days
2	10	3	1, 6 and 12 h; 1, 2, 3 and 5 days
3	10	2	1, 2, 3 days

**Table 2:** Variables of feeding behavior, monitored by electrical penetration graphs (EPGs), of *T. erytreae* species in citrus plants and carrot plants (means ± standard errors (SEs)) of the sequential and nonsequential variables.

Variable ^1^	Treat.	PPW	NWEI ^2^		WDI ^2^ (s)		WDEI ^2^ (s)	
			Mean ± SE	*p*	Mean ± SE	*p*	Mean ± SE	*p*
Time to 1st probe ^3^	Citrus	14/14			1649.7 ± 938.9	0.044		
	Carrot	11/11			9804.8 ± 3786.7			
No probe	Citrus	14/14	25.9 ± 4.7	0.002	14,342.0 ± 1655.9	0.002	1056.2 ± 362.5	<0.000
	Carrot	11/11	6.8 ± 1.9		23,769.7 ± 2051.2		10,748.4 ± 3608.2	
Probe	Citrus	14/14	25.3 ± 4.7	0.001	14,431.6 ± 1655.1	0.002		
	Carrot	8/11	5.9 ± 1.9		5030.3 ± 2051.2			
C	Citrus	14/14	25.5 ± 4.7	0.002	9647.6 ± 1350.5	0.001	782.0 ± 272.3	0.149
	Carrot	8/11	6.3 ± 1.9		3289.7 ± 1451.0		693.6 ± 465.5	
E1	Citrus	4/14	0.5 ± 0.3	0.244	20.3 ± 11.6	0.244	12.5 ± 6.8	0.244
	Carrot	0/11	0.0 ± 0.0		0.0 ± 0.0		0.0 ± 0.0	
E2	Citrus	3/14	0.4 ± 0.3	0.373	2494.5 ± 1392.2	0.373	1718.8 ± 1053.5	0.373
	Carrot	0/11	0.0 ± 0.0		0.0 ± 0.0		0.00 ± 0.00	
G	Citrus	6/14	0.4 ± 0.1	0.936	2269.3 ± 775.3	0.687	2269.3 ± 775.3	0.536
	Carrot	4/11	0.5 ± 0.2		1740.6 ± 881.1		1335.9 ± 600.4	
D	Citrus	6/14	0.6 ± 1.3	0.110	27.6 ± 67.2	0.169	13.1 ± 20.6	0.037
	Carrot	0/11	0.0 ± 0.0		0.0 ± 0.0		0.00 ± 0.00	
D with no E1	Citrus	2/14	0.1 ± 0.4	0.187	4.5 ± 12.9	0.244	4.5 ± 12.9	0.244
	Carrot	0/11	0.0 ± 0.0		0.0 ± 0.0		0.00 ± 0.00	

^1^ No probe: without activity. Probe: total count activity. C: activity in the intracellular space. E1: salivation in the phloem. E2: ingestion of the phloem. G: ingestion of the xylem. D: first contact with the phloem. ^2^ NWEI: number of waveform events per insect. WDI: waveform duration per insect. WDEI: duration of waveform event per insect. ^3^ Time to 1st probe from the start of the EPG.

**Table 3:** Results of CaLsol detection in *T. erytreae* specimens of each of the acquisition access periods (AAPs) evaluated.

Assay No.	AAPs							
	Control	1 h	6 h	12 h	1 Days	2 Days	3 Days	5 Days
1	0/40	- ^1^	-	-	9/40	-	-	-
2	0/30	0/30	0/30	2/30	1/30	5/30	9/30	10/30
3	0/20	-	-	-	3/20	5/20	14/20	-
Total ^2^	0%	0%	0%	6.70%	14.4%	20.0%	46.0%	33.3%

^1^ Not studied in this assay.^2^ Percentage of *T. erytreae* individuals positive for CaLsol in each AAP. The results include all the different replications together.

**Table 4:** Detection of CaLsol on *T. erytreae* individuals, carrot plants and citrus plants in restricted leaf exposure (clip cage plant) assays.

	IAP	Control	Carrot to Citrus ^2^	Control	Carrot to Carrot ^3^
Psyllids ^1^	24 h	0/22	16/76 (21.0%)	0/5	3/21 (14.3%)
	3 days	0/16	25/83 (30.1%)	0/4	3/9 (33.3%)
	7 days	0/9	10/59 (16.9%)	0/1	2/10 (20.0%)
	14 days	0/12	15/54 (27.8%)	0/5	0/8
Plants ^1^	24 h	0/6	0/14	0/2	0/6
	3 days	0/6	0/14	0/2	0/6
	7 days	0/6	0/14	0/2	1/6 (16.7%)
	14 days	0/6	0/13	0/2	1/6 (16.7%)

^1^ No. infected psyllids or plants/No. psyllids or plants tested. ^2^ From CaLsol-infected carrot plants to healthy citrus plants. ^3^ From CaLsol-infected carrot plants to healthy carrot plants.

## Supplementary Materials

The following are available online at https://www.mdpi.com/2075-4450/11/8/514/s1, Figure S1: Graph of the capture evolution of *Trioza erytreae* in sticky yellow traps (20 × 26 cm) placed above the canopy at 50 cm from the ground on a commercial carrot field where the distance between carrots and citrus plants was about 150 m. The carrot field was located in Tegueste (Tenerife, Spain). Traps were collected every week during 17 months. The capture evolution is shown as an average of the individuals caught by 9 sticky traps. Video S1: *Trioza erytreae* adult specimen, inserting its stylet into a grapevine (*Vitis vinifera* L.) leaf. Figure S2: Scheme of CaLsol transmission studies by dodder. Figure S3: Scheme of CaLsol transmission studies by budding (grafting). Figure S4: Scheme of CaLsol transmission studies by *T. erytreae*.
