# Supplementary figures and images for: ‘Candidatus Liberibacter Solanacearum’ Is Unlikely to Be Transmitted Spontaneously from Infected Carrot Plants to Citrus Plants by Trioza Erytreae
Source: Insects. 2020 Aug 8;11(8):514. doi: 10.3390/insects11080514 (PMC7469162; doi:10.3390/insects11080514)

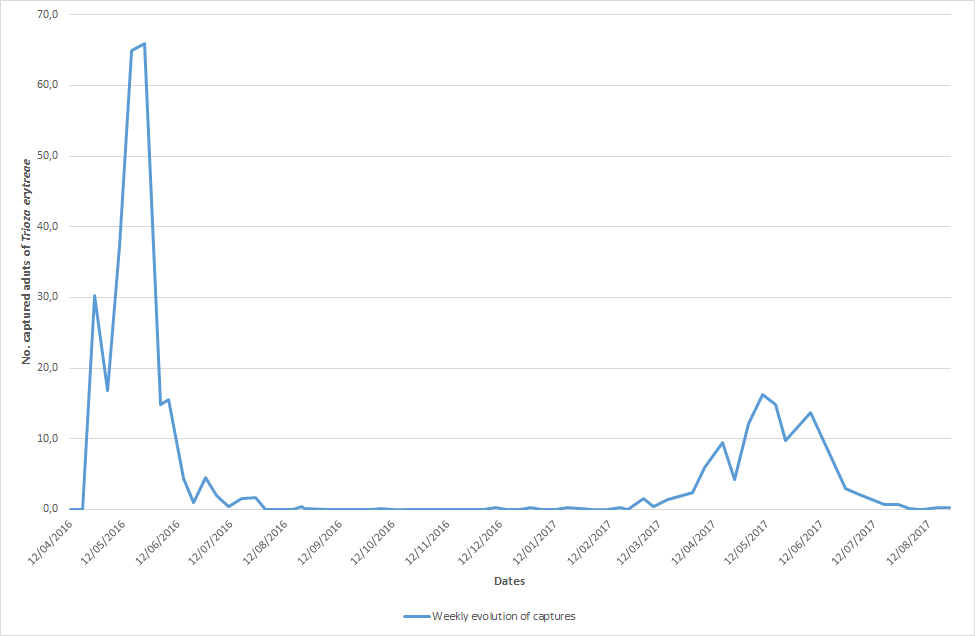

Supplement: Supplementary file 1 [file insects-11-00514-s001.zip › Figure S1.tif]

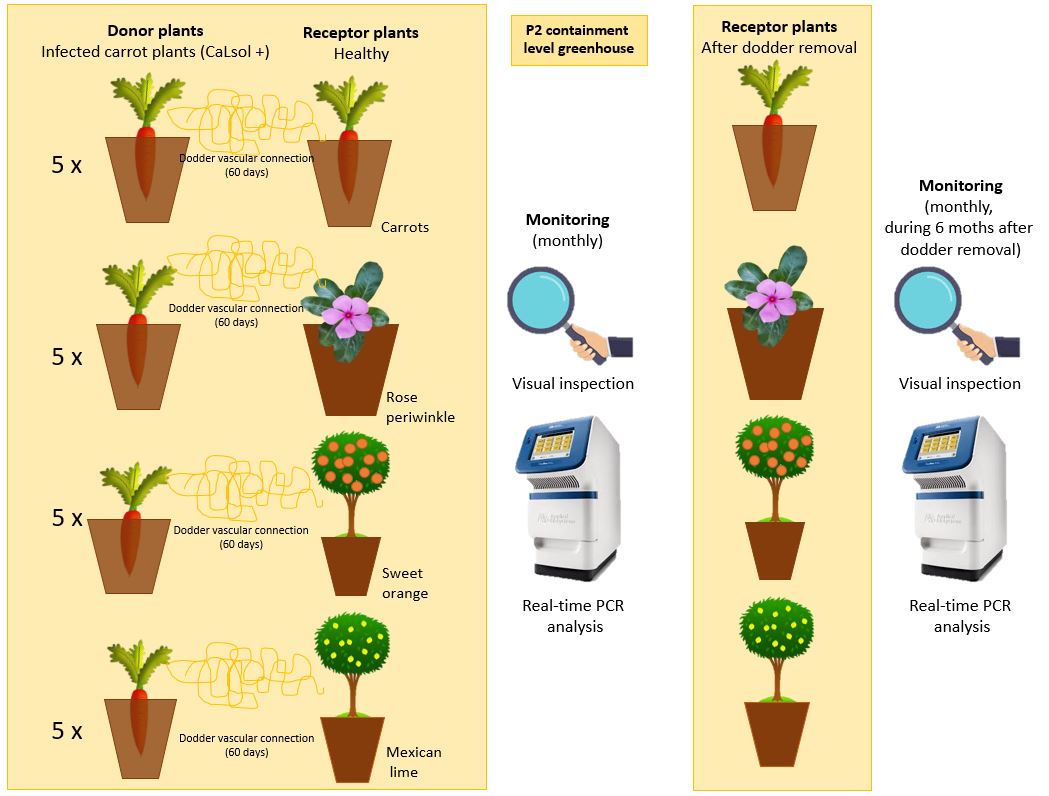

Supplement: Supplementary file 1 [file insects-11-00514-s001.zip › Figure S2.JPG]

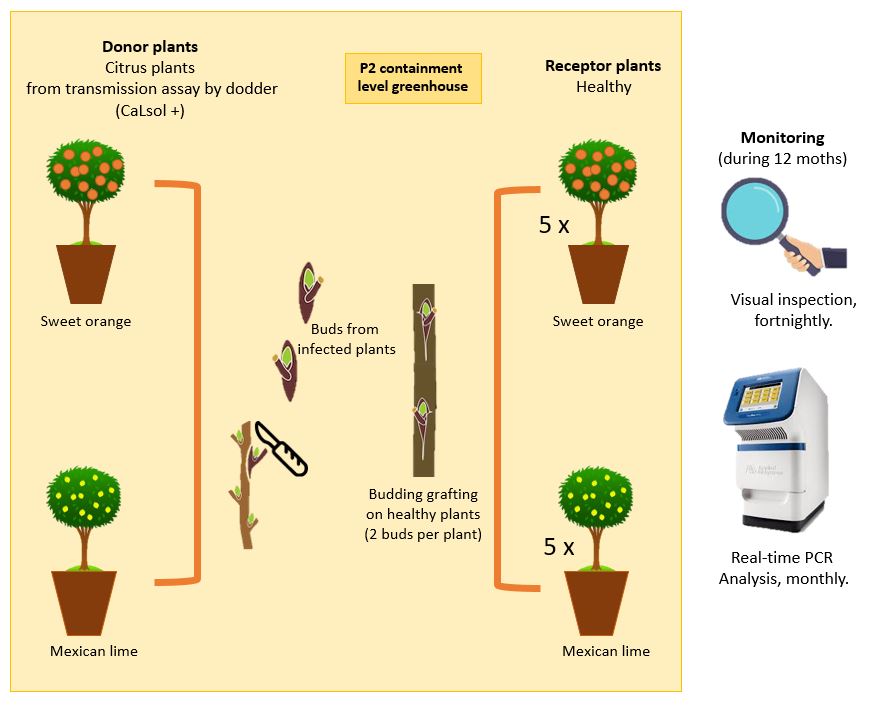

Supplement: Supplementary file 1 [file insects-11-00514-s001.zip › Figure S3.JPG]

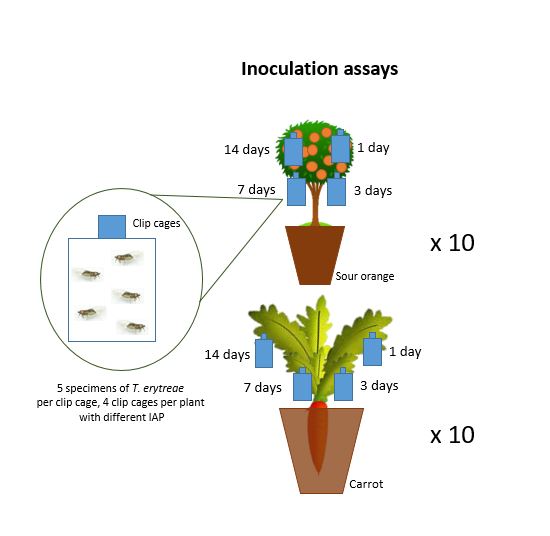

Supplement: Supplementary file 1 [file insects-11-00514-s001.zip › Figure S4.JPG]
